# Prevalence and Risk Factors for Poor Nutritional Status among Children in the Kilimanjaro Region of Tanzania

**DOI:** 10.3390/ijerph9103506

**Published:** 2012-10-05

**Authors:** Amina Abubakar, Jacqueline Uriyo, Sia E. Msuya, Mark Swai, Babill Stray-Pedersen

**Affiliations:** 1 Tilburg School of Social and Behavioral Sciences, Tilburg University, 5000 LE Tilburg, The Netherlands; 2 Division of Women and Children, Oslo University Hospital, Rikshospitalet and Institute of Clinical Medicine, University of Oslo, 0027 Oslo, Norway Email: jackieuriyo@yahoo.com (J.U.); babill.stray-pedersen@medisin.uio.no (B.S.-P.); 3 Kilimanjaro Christian Medical College, P.O. Box 3010, Moshi, Tanzania; Email: siamsuya@hotmail.com (S.E.M.); mswai@kcmc.ac.tz (M.S.)

**Keywords:** Africa, anaemia, children, SES, stunting, Tanzania, underweight

## Abstract

The current study investigated the prevalence and risk factors for poor nutritional status among children less than 36 months of age in the Kilimanjaro region of Tanzania. Using a cross sectional study design, children and their caregivers were recruited into the study. Anthropometric measures were taken based on established protocol while a standard questionnaire was utilized to collect socio-demographic data. A finger-prick blood sample was collected from all the children and haemoglobin (Hb) concentration analyzed using a HemoCue photometer (HemoCue AB, Angelholm, Sweden). Four hundred and twenty three (423) children (214 females) took part in this study. Participating children were aged between 1–35 months (mean = 13.04, SD = 7.70). We observed high rates of stunting (44.2%) and underweight (19.1%). Nearly 70% (n = 295) of the sample was anaemic (Hb < 11 g/dL). In a multivariate logistic regression model concerns on child growth, maternal education, and child’s age were found to independently predict stunting; whereas concerns over child’s growth and development, and distance to water source were found to uniquely predict being underweight. Maternal education was the only factor related to the child’s anaemia. The current study further emphasizes the need to implement context relevant interventions to combat malnutrition in this region of Tanzania and other similar settings.

## 1. Introduction

Malnutrition among children in developing countries is a major public health concern since it places a heavy burden on already disadvantaged communities. Poor physical growth, an indicator of poor nutritional status, is high in sub-Saharan countries, where approximately 21.9% of children are underweight and 40.1% are stunted [1]. The most vulnerable group of children are those under 5 years of age [2,3]. Anaemia, another indicator of poor nutritional status, is also widespread, with estimates indicating prevalence rates of 40–70% in Sub-Saharan countries. Both growth restriction and anemia in the early years of life increase the risk of mortality [4,5,6] and morbidity [7,8,9,10], and are associated with developmental and cognitive impairment [11,12,13,14,15]. 

The link between poverty and poor nutritional status among children has been widely reported [16,17]. Varying indicators of social economic status (SES) such as maternal and paternal educational level [18,19], parental income [20], and family assets such as the ownership of land, quality of housing, and foods harvested [21,22] among many SES indicators have all been associated with children’s nutritional status. Regardless of the method by which SES was estimated, its influence on child’s nutritional status was significant and consistent. It was observed that children from less advantaged families were more likely to experience growth restriction (*i.e.*, stunting and being underweight) compared to their peers from more advantaged backgrounds [13]. Despite this link several factors give compelling reasons to carry out further investigation into the relationship between SES and growth restriction. One of the most salient reasons for this is the fact that the prevalence of stunting and being underweight has been found to show both between- and within-country variation in sub-Saharan Africa [1,23,24]. There is thus a need examine prevalence and risk factors for poor growth outcomes in a variety of contexts so as to be able to plan targeted interventions. Compared to stunting and being underweight, relatively few studies have looked at the link between anaemia and social economic and demographic indicators, with most of the studies focusing on the relationship between anaemia and ill-health [25,26]. 

In this paper we report the prevalence and socioeconomic risk factors of stunting and being underweight among children aged 1–35 months in Same district, Kilimanjaro region, thus attempting to replicate earlier findings from other regions in Tanzania. Going beyond earlier findings, we evaluate the association between socioeconomic, demographic and health risk factors at the same time; a novel approach to studying the correlates of nutritional status in this region. Evaluating the different risk factors at the same time is significant since these variables are strongly correlated and have a synergic effect. Thus situating them together in the same model allows for the evaluation of the relative contribution of each in the growth status of the children. Moreover, we investigate the correlates to being anaemic and evaluate the relationship between anaemia and anthropometric status.

## 2. Methods

### 2.1. Study Sites

**Figure 1:**
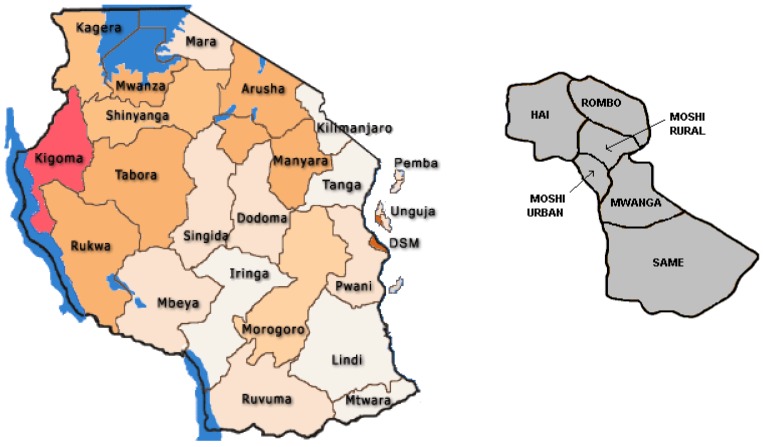
Map of the study area.

The study was conducted in Same (Figure 1), the largest of six districts in the Kilimanjaro region, in north Tanzania [25].Same District has an area of 5,186 km^2^ and is home to an estimated 254,549 people. Topographically the area is divided into three main zones, namely the Upland plateau zone which lies between an altitude of 1,100–2,462 m above the sea level, the Middle Plateau zone which lies between 900–1,100 m above sea level and the lowlands zone which rises from 500–900 m above the sea level. Administratively, the district it is divided into six divisions, 25 wards, one urban authority (same township), and 91 villages. About 70% of the population lives in the rural areas. The fertility rate is about 6 per woman and the birth rate is estimated at 28 per 1,000 population. Infant mortality rate is 8 deaths per 1,000 live births with the average household size ranging from 6–10 persons. The reported literacy rate and primary school enrolment is 98% respectively. About 80% of the total population depends on crop farming and livestock keeping as their main economic activities. The predominant ethnic group is the Pare, whose main language of communication is Kipare, while Kiswahili is used as the official language. In 2009 it was estimated that there were about 28,451 children aged 0–36 months.

### 2.2. Sampling Procedures

Village and street by single age population data from the 2002 census with 2009 projections were obtained from the Kilimanjaro regional bureau of statistics office in April 2009. A cluster sampling method with a probability proportional to size of the population was used to select 9 enumeration areas *i.e.*, villages or streets. To start with, the 0–36 month population for each village/street were listed with a column showing its cumulative population used as sampling frame. The sampling interval was then calculated: Total population of 0–36 month olds divided by the required number of clusters *i.e.*, 9. A random number between 0 and 1 was generated from the computer and the starting point for selection of the first cluster was determined by multiplying the random number with the sampling interval. The subsequent clusters were located by adding the sample interval to the previous number until the 10th cluster was selected. Compact segment sampling was used to select households within clusters. The selected clusters *i.e*., enumeration areas were mapped into segments with an approximately equal population. The number of segments was equal to the total population of 0–36 month olds divided by the cluster size *i.e.*, 9. Each segment included approximately 50 children. All the segments were assigned a number on pieces of paper and one was randomly picked. Within the selected segment, the study team visited all the households until 50 children whose parents had consented were examined. In cases where all the households in the segments had been surveyed and less than 50 children are available, a second segment was randomly selected. The members of the households in the selected segments were informed to be available for the survey at least one day before the survey. 

### 2.3. Measures

#### 2.3.1. Anthropometry

Height and weight measures were taken using recommended procedures [26]. The height of children younger than 24 months were measured lying down on the board (recumbent length), and standing height was measured for older children. Weights of undressed children were taken on a SECA Digital Scale. Height-for-Age (HAZ) and Weight-for-Age (WAZ) scores were generated using the WHO software for assessing growth and development [27]. All anthropometric measures were taken by two trained assistants. 

#### 2.3.2. Hb Levels

A finger-prick sample of blood was collected and haemoglobin concentration measured using a HemoCue photometer (HemoCue AB, Angelholm, Sweden).

#### 2.3.3. Social, Economic and Demographic Indicators

A standardized questionnaire was administered. The collected information can be categorized into three domains: parental, child and economic indicators. To evaluate *parental characteristics*, data was collected on parental age, marital status, educational level, occupation and maternal parity (*i.e.*, number of children born). Regarding *child characteristics*, we collected data on age in months, sex, birth weight and whether or not they were still breastfeeding. Additionally we asked the mother if the child had experienced any serious health problems since birth and if they (the mothers) were concerned about the child’s growth and development. To further evaluate *economic conditions* we recorded the kind of house they lived in (focusing on building materials used), whether they owned the house and the amount of time they used to go and fetch water. The use of ‘assets’ or ‘wealth index’ has been recommended as estimates of expenditure and income especially in developing countries where it is difficult to get reliable estimates of income [28,29]. 

**Table 1:** Background characteristics of the families sampled.

Background variables **		Number (Percentage)
Maternal Educational Level		
	No schooling	11 (2.6)
	Primary not completed	25 (5.9)
	Primary completed	352 (83.2)
	Secondary and above	35 (8.3)
Paternal Educational Level		
	No schooling	6 (1.5)
	Primary not completed	20 (4.9)
	Primary completed	334 (81.3)
	Secondary and above	51 (12.4)
Maternal Occupational level		
	Skilled	17 (4.1)
	Unskilled	389 (92.1)
	No occupation	9 (2.1)
	Student	7 (1.7)
Paternal Occupational level		
	Skilled	47 (11.0)
	Unskilled	365 (85.7)
	No occupation	1 (0.2)
	Student	5 (1.2)
Marital Status		
	Married	242(57.5)
	Single	67 (15.9)
	Divorced	10 (2.4)
	Widowed	3 (0.7)
	Cohabiting	99 (23.5)
Total number of maternal pregnancies		
	1	80 (19.2)
	2	98 (23.5)
	3	75 (18.0)
	4	52 (12.5)
	5	41 (9.8)
	6 and more	71 (17.0)
Is the child breastfeeding		
	Yes	332 (81.9)
	No	77 (18.4)
House Ownership		
	Yes	338 (83.9)
	No	65 (16.1)
Material used to build house		
	Mud	117(28.4)
	All other materials	295 (71.6)
Distance to water source		
	More than 15 minutes	141(34.9)
	Less than 15 minutes	263 (65.1)

** Numbers may be less than 423 in certain instances due to missing information.

Based on these questions a total of 19 potential predictors of child’s nutritional status were computed. Table 1 (above) and Table 2 (below) give a summary of how these variables were scored. 

**Table 2:** Characteristics of study participants.

Characteristic	(N = 423)
Mean Age in months (SD)	13.04 (7.78)
Boys (%)	209 (49.4%)
Incomplete immunization (%)	4 (0.9%)
Low birth weight **	16 (3.8%)
Concerns about child growth and development (%)	65 (15.4%)
Mother alive	422 (100%)
Father alive	420 (98.6%)
Mean Maternal age (SD)	28.67 (6.92)
Mean Paternal age (SD)	34.36 (8.11)

** 82 children (19.2%) were missing information on birth weight.

### 2.4. Data Management and Statistical Analysis

Data were analyzed using SPSS 17. Frequency tables and descriptive analysis were carried out to investigate the spread of scores. Univariate and Multivariate logistic regression were carried out to investigate the factors that predict poor growth outcomes. In separate models being underweight, stunting and being anaemic were included as dependent variables while parental characteristics, child characteristics and economic indicators were included as independent variables. The Odds Ratio (OR) and their 95% confidence interval (CI) were used to measure the strength of association between potential predictive factors and nutritional status. 

### 2.5. Ethical Considerations

The study was approved by the Kilimanjaro Christian Medical Centre Ethical Committee and Tanzanian National Institute of Medical Research (NIMR) and Norwegian Regional Ethical Committee. Written informed consent was sought from all study participants.

## 3. Results

### 3.1. Nutritional Status of the Sample

A total of 423 children aged 1–35 months took part in this study. Approximately 50% were male. Table 1 and Table 2 present the basic descriptors for this sample. Poor nutritional status was defined based on set standards. Stunting and being underweight were defined as having a z score below –2 *SD* of the WHO standards [30,31]. Based on these definitions, 44.2% (*N *= 187) and 19.1% (*N *= 81) were stunted and underweight, respectively; 67 of the children were both stunted and underweight. The mean HAZ and WAZ for this population were below the WHO standard, *M* = –1.90 (*SD* = 1.65, min: ‑11.22, max: 2.35) and *M* = –1.06 (*SD* = 1.17, min: –5.08, max: 2.63), respectively. No sex differences were observed in the WAZ and HAZ of the children in this population, *t*(421) = –1.63, *p *= 0.10 and *t*(421) = –0.042, *p *= 0.96. Children were classified as being mildly (Hb = 10.0–10.9 g/dL), moderately (Hb = 7.0–9.9 g/dL) or severely (Hb < 7.0 g/dL) anaemic [32]. Based on this definition 69.9% (*N *= 295), were observed to be anaemic, although most were mildly (126/422) or moderately (161/422) anaemic with only 2% (N = 8) being severely anaemic. No association was observed between sex and anaemia (χ^2 ^(1, *N* = 422) = 1.96, *p *= 0.16). Additionally we did not observe any association between the anthropometric variables and anaemia: being underweight (χ^2^ (1, *N* = 422) = 0.82, *p *= 0.36) and stunting (χ^2 ^(1, *N* = 422) = 0.14, *p *= 0.71). 

**Figure 2:**
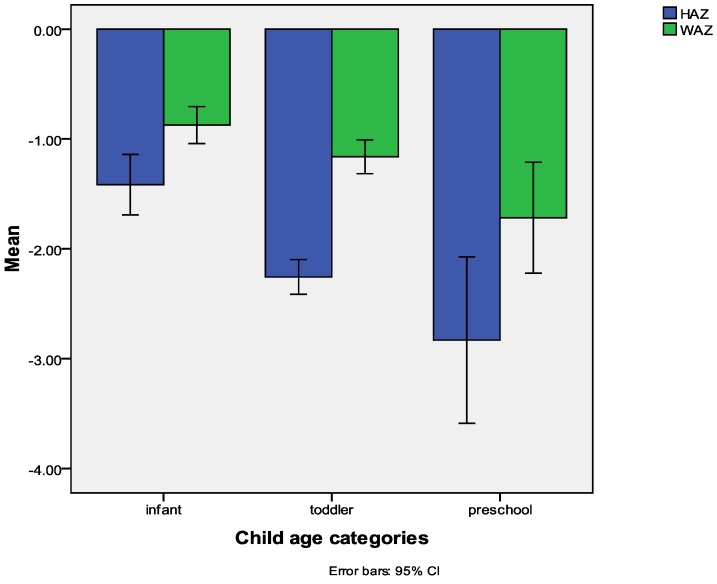
The rates of malnutrition per age group.

In this sample older children were at a significantly higher risk of being underweight or stunted. Figure 2 below indicates the mean HAZ and WAZ for children in different age categories; infants (2–12 months), toddlers (13–24 months) and preschoolers (25–35 months).

### 3.2. Predictors of Stunting

Significant predictors of stunting in a univariate logistic regression were maternal education (OR: 2.31 (95% CI: 1.47–3.64); *p* = 0.001), material used to build house (OR: 1.61 (95% CI: 1.05–2.48); *p* = 0.03), child age (OR: 0.91 (95% CI: 0.88–0.93); *p* = 0.001), serious childhood illness (OR: 0.51 (95% CI: 0.27–0.96); *p* = 0.038), concern over child’s growth and development (OR: 0.15 (95% CI: 0.08–0.30); *p* = 0.001), and breastfeeding (OR: 3.27(95% CI: 1.93–5.521); *p* < 0.001). In the multivariate analysis concerns on child growth, maternal education, and child’s age were found to independently predict stunting, Table 3 presents a summary of these results. 

**Table 3:** Multivariate analysis on risk factors for stunting.

Factors	B (SE)	Odds Ratio (95% C.I)
Materials used for building house	0.29 (0.25)	1.34 (0.82–2.12)
Concerns on child growth	–1.55 (0.37) ***	0.22 (0.10–0.44)
Child’s ill health	–0.34 (0.37)	0.71 (0.35–1.46)
Child age	–1.11 (0.22) ***	0.33 (0.21–0.51)
Maternal education	0.66 (0.25) **	1.94 (1.18–3.18)
Breastfed	0.28 (0.33)	1.32 (0.69–2.52)
Constant	0.79 (0.74)	

** *p *< 0.01, *** *p* < 0.001

### 3.3. Predictors of Being Underweight

Univariate logistic regression identified the paternal education (OR: 1.76 (95% CI: 1.07–2.89); *p* = 0.03), maternal age (OR: 0.95 (95% CI: 0.92–0.96); *p* = 0.005), paternal age (OR: 0.97 (95% CI: 0.94–0.99); *p* = 0.03), distance to water source (OR: 0.966 (95% CI: 0.936–0.997); *p* = 0.03), serious childhood illness (OR: 0.41 (95% CI: 0.20–0.80); *p* = 0.009), child’s age (OR: 0.96 (95% CI: 0.93–0.99); *p* = 0.016) number of maternal pregnancies (OR: 0.87 (95% CI: 0.76–0.99); *p* = 0.046) and concerns over child’s growth and development (OR:11.28 (95% CI: 6.247–20.39); *p* = 0.001) as risk factors for being underweight. Maternal education (OR: 1.56 (95% CI: 0.98–2.46); *p* = 0.057), was marginally significant and was included in the multivariate analysis; additionally low birth weight showed marginal significance (OR: 2.90 (95% CI: 1.12–7.44); *p* = 0.07), however the large number of missing values for birth weight (19.2% missing) and paternal age (10.9% missing) reduced the potential value as predictive factors and were therefore not included in the multivariate analysis. In the multivariate analysis reported concerns over child’s growth and development, and distance to water source were found to independently predict being underweight, see Table 4 for more details. 

**Table 4:** Multivariate analysis on risk factors of being underweight.

Factors	B (SE)	Odds Ratio (95% C.I)
Concerns on child growth	–2.76 (0.39) ***	0.06 (0.03–0.14)
Child’s ill health	-0.44 (0.45)	0.64 (0.27–1.55)
Maternal education	0.07 (0.32)	1.07 (0.56–2.01)
Child age	–0.26 (0.26)	0.76 (0.45–1.28)
Maternal age	–0.05 (0.03)	0.94 (0.89–1.01)
Distance to water source	–1.18 (0.37) **	0.31 (0.15–0.64)
Paternal education	0.29 (0.32)	1.32 (0.68–2.54)
Number of pregnancies	–0.05 (0.13)	0.94 (0.74–1.22)
Constant	4.16 (1.26)	

** *p* < 0.01, *** *p* < 0.001

### 3.4. Predictors of Being Anaemic

We carried out a series of univariate logistic regression involving all the 19 predictors (see Table 1 and Table 2). Results from this analysis indicate that maternal education (OR: 1.582 (95% CI: 1.05–2.40); *p* = 0.03) was the only factor that was significantly correlated to child anaemia. Given that only one variable was significant, no multivariate analysis was carried. 

## 4. Discussion

Our study indicates that there is a high prevalence of anaemia, stunting and being underweight among children in Kilimanjaro region of Tanzania. The high rates observed are similar to those observed elsewhere is East Africa, where up to 40% of the children have been observed to experience stunting [13]. The mean anthropometric status of this population is significantly below the WHO references populations which is generally consistent with findings from other parts of East Africa [13,33]. We observed that the risk factors for each of these nutritional deficiencies varied. For instance, stunting was closely associated with several sociodemographic factors in contrast to being underweight which was much more closely related to child’s health. These findings are consistent with the different aetiology of these two nutritional status indices and are consistent with earlier findings. HAZ is usually compromised following a period of chronic malnutrition, while being underweight is an indicator largely of short term nutritional compromise [2,31]. These results indicate the need to monitor different indicators of a child’s nutritional status as part of the health monitoring programmes to be able to provide adequate and timely intervention. 

Consistent with earlier reports we observed that age was a significant risk factor for poor physical growth [33]. These results emphasize the need to implement early intervention to reduce the number of children experiencing growth restriction. Moreover, the results further emphasized the need to investigate factors that contribute to continued growth faltering during toddlerhood. Regional based studies of factors contributing to early growth faltering may be salient since growth faltering has been associated with socio-cultural and feeding practices during the weaning period factors that are likely to vary on a regional basis.

We observed high rates of anaemia in our sample which is consistent with findings from other developing countries [34]. Moreover, the significant relationship between anaemia and maternal education has also been reported in earlier study from setting similar to ours [35,36]. Further investigation on the exact mechanism by which maternal education influence the child’s anaemic status is warranted. We hypothesize that maternal education may simply be a proxy for other factors such as childrearing practices, health seeking behaviour or feeding practices which significantly affect the children’s health. For instance, in Korea [36], it was observed that children of mothers who were better educated were less likely to be anaemic largely because better educated mothers were more likely to adopt healthier feeding habits.

Our results indicate that parental concerns of child’s growth and development was a strong predictor of child anthropometric status. These results have clear practical implications. The association between parental concerns and child anthropometric status provides validation for parental concerns and raise the question: to what extent can health workers utilize parental concerns as an indicator of child health? Can parental concerns be used as a screening instrument to identify children in need of closer monitoring, surveillance and intervention? In other fields dealing with child well-being, e.g., child development parental reports of concerns with child growth and development have been used to identify children who may be at-risk of poor developmental outcomes [37,38]. Based on these finding it can be recommended that as part of the post natal health services, health workers need to discuss and address parental concerns. This approach has two major benefits: a) it may potentially provide a quick and relatively cheap screening tool for health workers and b) allows the parent to become an active participant in monitoring the child’s health status. 

The most consistent indicator of child nutritional status was the maternal educational level; it was the only factor that was observed to significantly predict all the three nutritional indicators. The influence of maternal education could arise from various sources key amongst them is the impact it has on economic means (higher educated mothers have better jobs and more money) or more directly its influence on childrearing practices since mothers with more education have been observed to provide optimal care for their children. Regardless of the source of this influence our results further emphasize the need to invest in girls’/mothers’ education as a way to enhance child wellbeing in developing countries [39,40]. 

The current study adds to the body of knowledge linking social, economic and demographic factors to the nutritional status of children in different settings. However, this study is cross-sectional in nature therefore limits our ability to make inferences on causation. Additionally, we did study other potentially salient predictors of nutritional outcomes. For instance we did not evaluate the impact of ill-health on nutritional status. Yet, anaemia has been strongly linked to ill health and especially helminthic infections [41] and malaria particularly in malaria endemic areas [42]. This forms another limitation of our study. 

## 5. Conclusions

In conclusion, we observed that high rates of nutritional deficiency and these deficiencies were related to maternal, individual and household characteristics. However, the most consistent risk factor was observed to be maternal education further emphasizing the need to invest in women education as an intervention for enhancing child wellbeing. The current study further emphasizes the need to implement context relevant interventions to combat malnutrition in this region of Tanzania and other similar settings. 
